# Mitochondrial genomic variation in dementia with Lewy bodies: association with disease risk and neuropathological measures

**DOI:** 10.1186/s40478-022-01399-4

**Published:** 2022-07-14

**Authors:** Rebecca R. Valentino, Chloe Ramnarine, Michael G. Heckman, Patrick W. Johnson, Alexandra I. Soto-Beasley, Ronald L. Walton, Shunsuke Koga, Koji Kasanuki, Melissa E. Murray, Ryan J. Uitti, Julie A. Fields, Hugo Botha, Vijay K. Ramanan, Kejal Kantarci, Val J. Lowe, Clifford R. Jack, Nilufer Ertekin-Taner, Rodolfo Savica, Jonathan Graff-Radford, Ronald C. Petersen, Joseph E. Parisi, R. Ross Reichard, Neill R. Graff-Radford, Tanis J. Ferman, Bradley F. Boeve, Zbigniew K. Wszolek, Dennis W. Dickson, Owen A. Ross

**Affiliations:** 1 Department of Neuroscience, Mayo Clinic, 4500 San Pablo Road, Jacksonville, FL 32224 USA; 2 Division of Biomedical Statistics and Informatics, Mayo Clinic, Jacksonville, FL 32224 USA; 3 Department of Neuropsychiatry, St. Marianna University School of Medicine, Kanagawa, Japan; 4 Department of Neurology, Mayo Clinic, Jacksonville, FL 32224 USA; 5 Department of Psychiatry and Psychology, Mayo Clinic, Rochester, MN 55905 USA; 6 Department of Neurology, Mayo Clinic, Rochester, MN 55905 USA; 7 Department of Radiology, Mayo Clinic, Rochester, MN 55905 USA; 8 Laboratory Medicine and Pathology, Mayo Clinic, Rochester, MN 55905 USA; 9 Department of Psychiatry and Psychology, Mayo Clinic, Jacksonville, FL 32224 USA; 10 Department of Clinical Genomics, Mayo Clinic, Jacksonville, FL 32224 USA

**Keywords:** Dementia with Lewy-bodies, Lewy body disease, Mitochondrial DNA, Mitochondrial haplogroups, Neuropathology

## Abstract

Dementia with Lewy bodies (DLB) is clinically diagnosed when patients develop dementia less than a year after parkinsonism onset. Age is the primary risk factor for DLB and mitochondrial health influences ageing through effective oxidative phosphorylation (OXPHOS). Patterns of stable polymorphisms in the mitochondrial genome (mtDNA) alter OXPHOS efficiency and define individuals to specific mtDNA haplogroups. This study investigates if mtDNA haplogroup background affects clinical DLB risk and neuropathological disease severity. 360 clinical DLB cases, 446 neuropathologically confirmed Lewy body disease (LBD) cases with a high likelihood of having DLB (LBD-hDLB), and 910 neurologically normal controls had European mtDNA haplogroups defined using Agena Biosciences MassARRAY iPlex technology. 39 unique mtDNA variants were genotyped and mtDNA haplogroups were assigned to mitochondrial phylogeny. Striatal dopaminergic degeneration, neuronal loss, and Lewy body counts were also assessed in different brain regions in LBD-hDLB cases. Logistic regression models adjusted for age and sex were used to assess associations between mtDNA haplogroups and risk of DLB or LBD-hDLB versus controls in a case-control analysis. Additional appropriate regression models, adjusted for age at death and sex, assessed associations of haplogroups with each different neuropathological outcome measure. No mtDNA haplogroups were significantly associated with DLB or LBD-hDLB risk after Bonferroni correction.Haplogroup H suggests a nominally significant reduced risk of DLB (OR=0.61, P=0.006) but no association of LBD-hDLB (OR=0.87, P=0.34). The haplogroup H observation in DLB was consistent after additionally adjusting for the number of *APOE* ε4 alleles (OR=0.59, P=0.004). Haplogroup H also showed a suggestive association with reduced ventrolateral substantia nigra neuronal loss (OR=0.44, P=0.033). Mitochondrial haplogroup H may be protective against DLB risk and neuronal loss in substantia nigra regions in LBD-hDLB cases but further validation is warranted.

**Supplementary Information:**

The online version contains supplementary material available at 10.1186/s40478-022-01399-4.

## Introduction

Lewy body dementia are comprised of two distinct, but clinically related, disorders—Dementia with Lewy bodies (DLB) and Parkinson’s disease dementia (PDD) [22, 30]. The timing of dementia onset determines the exact clinical diagnosis, whereby dementia onset before or less than a year after parkinsonism is classified as DLB, and dementia onset more than one year after parkinsonism is classified as PDD [30]. DLB is one of the most common forms of dementia after Alzheimer’s disease (AD), accounting for approximately 23% of all dementia cases [49]. Currently there is no treatment to prevent or cure DLB and disease course is progressive and eventually fatal.

Neuropathologically, DLB and PDD are very similar and fall under the pathological term of Lewy body disease (LBD). Lewy body disorders are characterized by the presence of Lewy bodies (LB) and Lewy neurites in the brain, causing neurodegeneration. LB are complex masses of aggregated phosphorylated alpha-synuclein (aSyn), p62, and ubiquitin proteins, as well as lipids and membranous organelles [29]. The location and distribution of LBs in the brain and the associated neuronal dysfunction determines clinical phenotypes observed. For example, LB accumulation in the brainstem and midbrain regions, and the associated neurodegeneration, typically induces Parkinson’s disease (PD) symptoms of tremor, rigidity, and slowness of movement [38], whereas LB accumulation in the neocortical and limbic regions is associated with cognitive and neuropsychiatric symptoms, such as cognitive impairment, fluctuations, visual hallucinations, and behavioral changes—which are reflective of PDD or DLB [12, 30, 37]. Classical brainstem and nigral LB consist of dense, spherical cores with irradiating filaments, and a surrounding halo (when stained with hematoxylin/eosin), whereas LB in neocortical regions typically have pale, fibrillary structures without a halo or central core [29, 42]. Paler, fibrillary LB have been described as premature and are thought to develop into classical LB structures with disease progression [17]. In addition to LB, pathological aggregates of extracellular amyloid-beta (Abeta) plaques and intracellular neurofibrillary tangles of hyperphosphorylated tau proteins are often present in LBD making the disease spectrum very heterogenous [10]. Neuropathologists use defined criteria to assess aSyn and tau Braak stage, as well as beta-amyloid Thal phase, to neuropathologically determine accurate LBD diagnosis and characterize disease severity, and use available medical records to determine the likelihood of clinical phenotypes [6].

Within the past decade, ongoing efforts have continued to work towards understanding genetic markers influencing LB disorders, particularly PD, whereby current case-control studies consist of tens of thousands of cases [18, 34, 35]. Recent smaller case-control studies of DLB have identified overlapping genetic markers between PD and AD [3, 18, 27], further demonstrating the overlapping pathologies of these diseases, but despite such efforts, the genetic etiology of DLB is yet to be defined. Thus, providing additional scope to characterize other genetic factors which may be driving dementia onset in DLB.

Age consistently remains the major risk factor for neurodegeneration and both healthy ageing and aSyn accumulation is influenced by mitochondrial health, whereby increased reactive oxygen species (ROS) production accelerates ageing and aSyn aggregation over time [20, 28]. ROS are a byproduct from oxidative phosphorylation (OXPHOS) which occurs on the inner mitochondrial membrane [26]. Mitochondria contain their own genomic information (mtDNA), independent to the nuclear genome, which codes for 13 essential subunits in OXPHOS complexes. Patterns of stable polymorphisms across the mtDNA molecule define individuals to specific mtDNA haplogroups, and each mtDNA haplogroup has a unique metabolic profile which influences ROS production over time [15, 16]. As a result of their distinct metabolic backgrounds, mtDNA haplogroups have been associated with age-related and multiple neurodegenerative diseases, including PD and AD [4, 21], but have not been examined in relation to dementia onset in large cohorts of patients. Therefore, the aims of this study were to evaluate the association between mtDNA haplogroups and risk of clinical DLB and pathologically confirmed LBD cases with a high likelihood of having clinical DLB (LBD-hDLB) in a case-control analysis. In analysis of the LBD-hDLB group, we also examined associations of mtDNA haplogroups with severity of neuropathological measures, such as LB counts and distribution, neuronal loss, and dopaminergic degeneration across several brain regions.

## Material and methods

### Study subjects and data collection

A total of 806 DLB subjects (N=360 clinically diagnosed DLB cases and N=446 autopsy-confirmed Lewy body disease cases that were assessed as having a high likelihood of DLB (LBD-hDLB) – of which N=48 were present in both series) and 910 controls were included in this study. Clinical DLB patients were diagnosed by neurologists at Mayo Clinic in Jacksonville, FL or Rochester, MN and were recruited as part of the Alzheimer’s Disease Research Center and the Mayo Clinic Study of Aging. Pathologically confirmed LBD cases were obtained from the brain bank for neurodegenerative disorders at Mayo Clinic in Jacksonville, FL and were evaluated by a single neuropathologist (Dr. Dennis Dickson). LBD cases were all assessed as having a high likelihood of DLB according to the criteria of the fourth report of the DLB consortium [30]. Controls were recruited by Dr. Zbigniew Wszolek and his colleagues from Mayo Clinic in Jacksonville, FL and were absent of neurological disease. All subjects provided written consent prior to study commencement and were Caucasian, non-Hispanic, and unrelated. Age at DLB diagnosis in clinically diagnosed DLB cases, age at death in pathologically confirmed LBD-hDLB cases, and age at blood draw in controls, and sex was collected for all subjects (Table 1). Additionally, neuropathological measures for Lewy body counts and substantia nigra (SN) neuronal loss were available for 242 (54.3%) LBD-hDLB cases (Table 1).

**Table 1:** Patient characteristics

Variable	Controls (N = 910)	Clinical DLB (N = 360)	LBD with a high likelihood of DLB (N = 446)
Age (years)^a^	79 (41, 102)	73 (50, 100)	78 (48, 103)
*Sex*			
Male	388 (42.6%)	270 (75.0%)	292 (65.5%)
Female	522 (57.4%)	90 (25.0%)	154 (34.5%)
*Braak NFT stage*			
0	N/A	N/A	13 (2.9%)
I	N/A	N/A	23 (5.2%)
II	N/A	N/A	138 (30.9%)
III	N/A	N/A	143 (32.1%)
IV	N/A	N/A	129 (28.9%)
V	N/A	N/A	0 (0.0%)
VI	N/A	N/A	0 (0.0%)
*Thal amyloid phase*			
0	N/A	N/A	40 (10.7%)
1	N/A	N/A	37 (9.9%)
2	N/A	N/A	23 (6.1%)
3	N/A	N/A	129 (34.4%)
4	N/A	N/A	56 (14.9%)
5	N/A	N/A	90 (24.0%)
*LBD subtype*			
Transitional	N/A	N/A	89 (20.0%)
Diffuse	N/A	N/A	357 (80.0%)
*Lewy body counts*			
Middle frontal gyrus	N/A	N/A	5 (0, 35)
Superior temporal gyrus	N/A	N/A	10 (0, 50)
Inferior parietal gyrus	N/A	N/A	4 (0, 30)
Cingulate gyrus	N/A	N/A	12 (2, 32)
Parahippocampal gyrus	N/A	N/A	16 (1, 45)
*Putaminal TH-ir*			
Dorsolateral	N/A	N/A	2.93 (0.26, 21.61)
Ventromedial	N/A	N/A	8.99 (0.26, 27.42)
*Substantia nigra neuronal loss score*			
Ventrolateral			
0.0 = none	N/A	N/A	0 (0.0%)
0.5 = none/mild	N/A	N/A	2 (1.0%)
1.0 = mild	N/A	N/A	10 (5.1%)
1.5 = mild/moderate	N/A	N/A	7 (3.6%)
2.0 = moderate	N/A	N/A	21 (10.8%)
2.5 = moderate/severe	N/A	N/A	23 (11.8%)
3.0 = severe	N/A	N/A	132 (67.7%)
Medial			
0.0 = none	N/A	N/A	2 (1.1%)
0.5 = none/mild	N/A	N/A	1 (0.6%)
1.0 = mild	N/A	N/A	25 (14.0%)
1.5 = mild/moderate	N/A	N/A	16 (8.9%)
2.0 = moderate	N/A	N/A	26 (14.5%)
2.5 = moderate/severe	N/A	N/A	25 (14.0%)
3.0 = severe	N/A	N/A	84 (46.9%)

Sample median (minimum, maximum) is given for continuous variables

For LBD cases with a high likelihood of DLB, information was unavailable for Thal amyloid phase (N = 71), middle frontal gyrus Lewy body count (N = 211), superior temporal gyrus Lewy body count (N = 213), inferior parietal gyrus Lewy body count (N = 211), cingulate gyrus Lewy body count (N = 213), parahippocampal gyrus Lewy body count (N = 232), dorsolateral putaminal TH-ir (N = 250), ventromedial putaminal TH-ir (N = 250), ventrolateral substantia nigra neuronal loss score (N = 251), and medial substantia nigra neuronal loss score (N = 267).

^a^Age represents age at blood draw for controls, age at DLB onset for clinical DLB cases, and age at death for LBD with a high likelihood of DLB cases

### Neuropathological assessment in LBD-hDLB

#### Assessment of neurofibrillary tangles, senile plaques, and Lewy bodies

Neuropathological methodologies used to assess neurofibrillary tangles (NFTs), senile plaques (SPs), and Lewy bodies (LBs) have been described previously [33]. Briefly, neuroanatomical sampling and thioflavin-S fluorescence microscopy was performed, where counts of NFTs and SPs were measured manually in six cortical regions, four sections of the hippocampus, and two regions of the amygdala [43]. Formalin-fixed, paraffin-embedded tissue samples from limbic and cortical regions were sectioned and mounted on glass slides. Assessment of LB pathology was performed using an aSyn antibody (NACP, 1:3000 rabbit polyclonal, Mayo Clinic antibody) with formic acid pretreatment for 30 minutes and was processed using the DAKO Autostainer (DAKO Auto Machine Corporation, Carpinteria, CA) with DAKO Envision+ HRP System. LB counts were measured in five cortical regions—middle frontal, superior temporal, inferior parietal, cingulate, and parahippocampal. The distribution of LB pathology was assessed using the staging scheme defined by Kosaka *et al.* to categorize samples as either brainstem, transitional, or diffuse [25]. Braak NFT stage [1] and Thal amyloid phase [44] were assigned according to the distributions of NFTs and SPs respectively. These neuropathologic measures are summarized in Table 1.

#### Quantification of striatal dopaminergic degeneration

Quantitative assessment of striatal dopaminergic degeneration by measurement of tyrosine hydroxylase immunoreactivity (TH-ir) has been described previously [24]. To summarize, the putamen was assessed at the level of the anterior commissure from a section made from the hemi-brain in a standardized dissection plane defined by three points in the fundibulum, uncus, and posterior margin of the anterior commissure in the third ventricle. Digital images of the putamen were parcelled into ventromedial and dorsolateral areas [19], and dopaminergic degeneration was quantitatively assessed.

The basal ganglia section was processed for immunohistochemistry with a commercially available antibody to TH (rabbit polyclonal, 1:600; Affinity Bioreagants, Golden, Colorado) with Proteinase K pretreatment for 5 minutes. The immunostained sections were captured by ScanScope XT (Aperio Technologies, Vista, California), and images were annotated with ImageScope (version 12.1). Regions of interest were manually edited to exclude artifacts, large blood vessels and their perivascular spaces, and large fiber bundles. The putamen was divided into ventromedial and dorsolateral regions. Quantification of TH-ir used an algorithm that detected positive pixels based on optical density. TH-ir was expressed as a percentage, calculated as the number of positive pixels divided by the sum of inverse pixels and background pixels. A lower TH-ir value represents a greater degree of putaminal dopaminergic degeneration. Table 1 summarizes dorsolateral and ventromedial putaminal TH-ir in DLB cases.

#### Assessment of substantia nigra pigmented neuronal loss

The midbrain was a transverse section at the level of the third nerve, similar to what has been recommended for diagnostic evaluation of PD [7]. A semi-quantitative assessment of SN cell groups was ascertained on hematoxylin and eosin-stained sections at 100x magnification. Our assessment was restricted to pigmented neurons of SN pars compacta and divided into medial and ventrolateral sections—similar to previous studies [14, 39]. We used a human atlas of SN cell groups to identify medial and ventrolateral regions of the SN [36]. The density of nonpigmented neurons was not taken into consideration for the assessment of the semi-quantitative scores, which were based on a 4-point scale (0=none, 1=mild, 2=moderate, and 3=severe) (Table 1).

### Genetic analysis

Peripheral blood was collected from clinical DLB patients and control subjects, and frozen cerebellum brain tissue was provided from pathologically confirmed LBD-hDLB cases. Genomic DNA was extracted from peripheral blood lymphocytes and cerebellum tissue using Autogen Flex Star and Autogen 245T (Holliston, MA) methods respectively. DNA was diluted to 15 ng/µl and 39 unique mitochondrial DNA variants were genotyped by a single-user (RRV) using two custom-designed Agena Bioscience iPLEX arrays on Sequenom MassARRAY technology [11]. More detailed methods for genetic assessments have been previously published [46, 47]. Individual mitochondrial DNA haplogroups were defined to mitochondrial phylogeny for each subject [48] (Table 2). Phylogenetically related haplogroups were also grouped into family haplogroups (e.g. sub-haplogroups H, H1, H2, H3, and H4 are all part of the family H haplogroup) and four different super-haplogroups (e.g. family J and family T haplogroups are super-haplogroup JT) for secondary analysis assessments. Haplogroups that occurred in fewer than 10 subjects in a given association analysis were not analyzed in that specific analysis. All cases were examined for population stratification prior to conducting this study [3].

**Table 2:** Associations between mitochondrial DNA haplogroups and risk of DLB

Mitochondrial DNA Haplogroup	Haplogroup frequency, No. (%)				Clinical DLB vs. controls		LBD with a high likelihood of DLB vs. controls		Overall DLB vs. controls	
	Controls (N = 910)	Clinical DLB (N = 360)	LBD high likelihood of DLB (N = 446)	Overall DLB (N = 758)	OR (95% CI)	*P*-value	OR (95% CI)	*P*-value	OR (95% CI)	*P*-value
N^a^	2 (0.2%)	0 (0.0%)	0 (0.0%)	0 (0.0%)	–	–	–	–	–	–
N1^a^	5 (0.5%)	4 (1.1%)	2 (0.4%)	6 (0.8%)	–	–	–	–	1.31 (0.38, 4.49)	0.66
I	31 (3.4%)	12 (3.3%)	13 (2.9%)	22 (2.9%)	0.77 (0.38, 1.56)	0.47	0.83 (0.41, 1.60)	0.60	0.77 (0.44, 1.38)	0.38
W	15 (1.6%)	9 (2.5%)	8 (1.8%)	15 (2.0%)	1.71 (0.69, 4.23)	0.24	1.11 (0.44, 2.64)	0.81	1.30 (0.61, 2.76)	0.50
X	8 (0.9%)	2 (0.6%)	7 (1.6%)	9 (1.2%)	0.60 (0.12, 3.10)	0.55	1.68 (0.57, 4.82)	0.33	1.36 (0.50, 3.69)	0.55
R or R0^a^	6 (0.7%)	1 (0.3%)	1 (0.2%)	2 (0.3%)	–	–	–	–	–	–
HV or HV0a	22 (2.4%)	6 (1.7%)	13 (2.9%)	17 (2.2%)	0.86 (0.33, 2.26)	0.76	1.36 (0.65, 2.74)	0.40	1.02 (0.53, 2.00)	0.94
H, H1, H2, H3, and H4	423 (46.5%)	149 (41.4%)	183 (41.0%)	316 (41.7%)	0.85 (0.65, 1.10)	0.22	0.83 (0.66, 1.05)	0.13	0.85 (0.70, 1.04)	0.12
H	199 (21.9%)	51 (14.2%)	85 (19.1%)	133 (17.5%)	0.61 (0.43, 0.87)	0.006	0.87 (0.65, 1.16)	0.34	0.78 (0.61, 1.01)	0.057
H1	145 (15.9%)	67 (18.6%)	56 (12.6%)	116 (15.3%)	1.22 (0.87, 1.71)	0.26	0.78 (0.55, 1.09)	0.16	0.97 (0.74, 1.28)	0.85
H2	36 (4.0%)	6 (1.7%)	12 (2.7%)	17 (2.2%)	0.44 (0.18, 1.09)	0.076	0.67 (0.33, 1.28)	0.24	0.56 (0.30, 1.02)	0.057
H3	32 (3.5%)	16 (4.4%)	23 (5.2%)	36 (4.7%)	1.42 (0.73, 2.75)	0.30	1.59 (0.89, 2.78)	0.11	1.48 (0.89, 2.47)	0.13
H4	11 (1.2%)	9 (2.5%)	7 (1.6%)	14 (1.8%)	1.76 (0.70, 4.46)	0.23	1.14 (0.41, 2.98)	0.80	1.33 (0.59, 3.01)	0.50
V	18 (2.0%)	14 (3.9%)	12 (2.7%)	26 (3.4%)	1.99 (0.94, 4.22)	0.073	1.37 (0.63, 2.91)	0.41	1.82 (0.97, 3.42)	0.064
JT^a^	2 (0.2%)	0 (0.0%)	0 (0.0%)	0 (0.0%)	–	–	–	–	–	–
J, J1, J1d, J2, J2a, and J2b	93 (10.2%)	38 (10.6%)	52 (11.7%)	87 (11.5%)	1.02 (0.67, 1.55)	0.94	1.13 (0.78, 1.63)	0.51	1.11 (0.81, 1.54)	0.51
J	0 (0.0%)	1 (0.3%)	0 (0.0%)	1 (0.1%)	–	–	–	–	–	–
J1	72 (7.9%)	25 (6.9%)	41 (9.2%)	64 (8.4%)	0.82 (0.50, 1.35)	0.44	1.11 (0.73, 1.67)	0.61	1.02 (0.71, 1.46)	0.93
J1d	1 (0.1%)	1 (0.3%)	0 (0.0%)	1 (0.1%)	–	–	–	–	–	–
J2a	13 (1.4%)	7 (1.9%)	9 (2.0%)	15 (2.0%)	1.80 (0.67, 4.89)	0.25	1.74 (0.70, 4.16)	0.22	1.66 (0.76, 3.62)	0.20
J2b	7 (0.8%)	4 (1.1%)	2 (0.4%)	6 (0.8%)	1.20 (0.32, 4.48)	0.79	–	–	0.95 (0.30, 2.95)	0.92
T, T1, and T2	77 (8.5%)	32 (8.9%)	45 (10.1%)	71 (9.4%)	1.16 (0.73, 1.84)	0.53	1.25 (0.84, 1.86)	0.26	1.17 (0.83, 1.67)	0.38
T^a^	0 (0.0%)	1 (0.3%)	0 (0.0%)	1 (0.1%)	–	–	–	–	–	–
T1	17 (1.9%)	5 (1.4%)	8 (1.8%)	13 (1.7%)	0.64 (0.22, 1.85)	0.41	0.85 (0.34, 1.96)	0.71	0.83 (0.39, 1.76)	0.62
T2	60 (6.6%)	26 (7.2%)	37 (8.3%)	57 (7.5%)	1.27 (0.76, 2.12)	0.35	1.38 (0.89, 2.14)	0.15	1.25 (0.84, 1.85)	0.26
U, U1, U3, U5, U6, and U8b'c	130 (14.3%)	53 (14.7%)	67 (15.0%)	110 (14.5%)	0.97 (0.67, 1.40)	0.89	1.01 (0.73, 1.40)	0.95	0.96 (0.72, 1.28)	0.79
U	44 (4.8%)	22 (6.1%)	21 (4.7%)	40 (5.3%)	1.12 (0.64, 1.96)	0.68	0.89 (0.51, 1.51)	0.66	0.97 (0.61, 1.52)	0.88
U1^a^	1 (0.1%)	0 (0.0%)	2 (0.4%)	2 (0.3%)	–	–	–	–	–	–
U3^a^	8 (0.9%)	0 (0.0%)	1 (0.2%)	1 (0.1%)	–	–	–	–	–	–
U5	74 (8.1%)	30 (8.3%)	42 (9.4%)	65 (8.6%)	0.99 (0.62, 1.59)	0.97	1.13 (0.74, 1.68)	0.57	1.02 (0.71, 1.47)	0.90
U6^a^	3 (0.3%)	1 (0.3%)	0 (0.0%)	1 (0.1%)	–	–	–	–	–	–
U8b'c^a^	0 (0.0%)	0 (0.0%)	1 (0.2%)	1 (0.1%)	–	–	–	–	–	–
K	78 (8.6%)	40 (11.1%)	43 (9.6%)	77 (10.2%)	1.23 (0.80, 1.88)	0.35	1.12 (0.74, 1.66)	0.59	1.15 (0.81, 1.62)	0.44

OR = odds ratio; CI = confidence interval. ORs, 95% CIs, and p-values result from logistic regression models that were adjusted for age and sex. The 47 cases that were in both the clinical DLB series and the LBD with a high likelihood of DLB series were included only once in the overall DLB series. After applying a Bonferroni correction for multiple testing, *P*-values <  0.0023 (clinical DLB vs. controls), <  0.0024 (LBD with a high likelihood of DLB vs. controls), and <  0.0022 (overall DLB vs. controls) were considered as statistically significant.

^a^Haplogroups that occurred in < 10 subjects in a given association analysis were not examined.

### Statistical analysis

Associations of mitochondrial haplogroups with risk of clinical DLB and LBD-hDLB, each separately versus controls, were examined using logistic regression models that were adjusted for age and sex. Odds ratios (ORs) and 95% confidence intervals (CIs) were estimated. Additionally, clinical DLB and LBD-hDLB series were combined into one overall DLB series, and associations of haplogroups with risk of DLB in comparison to controls were assessed. The 48 cases that were present in both the clinical DLB series and the LBD-hDLB series, were only included once in the overall DLB series.

In the LBD-hDLB series, associations of haplogroups with each different neuropathological outcome measure were assessed using age at death and sex-adjusted regression models that are appropriate for the nature of the given outcome measure. Specifically, associations of haplogroups with dorsolateral and ventromedial putaminal TH-ir were examined using linear regression models, where due to their skewed distributions, lateral putaminal TH-ir was considered on the logarithm (base-10) scale and medial putaminal TH-ir was considered on the square root scale. Regression coefficients and 95% CIs were estimated and are interpreted as the additive increase on the mean outcome measure (on the logarithm or square root scale) for the given haplogroup. Associations of haplogroups with ventrolateral and medial SN neuronal loss scores were assessed using proportional odds logistic regression models. ORs and 95% CIs were estimated and are interpreted at the multiplicative increase on the odds or a more severe neuronal loss score for the given haplogroup. Neuronal loss scores ≤1 (ventrolateral) and ≤1.5 (medial) were combined into one category in proportional odds logistic regression analysis due to their low frequencies. Associations between haplogroups and cortical LB counts were evaluated using negative binomial regression models. Multiplicative effects and 95% CIs were estimated and are interpreted as the multiplicative increase on the mean LB count for the given haplogroup. Finally, binary logistic regression models were used to assess associations between haplogroups and LBD subtype. ORs and 95% CIs for presence of diffuse LBD were estimated.

We utilized a Bonferroni correction for multiple testing separately for each outcome measure in the primary analysis that did not involve super-haplogroups (*P*-values <0.05 were considered statistically significant in secondary super-haplogroup analysis). As haplogroups that occurred in less than 10 subjects in a given association analysis were not analyzed in that specific analysis, and the degree of missing data differed between outcomes, the Bonferroni-corrected statistical significance level correspondingly varied between outcomes (see table footnotes for details). All statistical tests were two-sided. Statistical analyses were performed using R Statistical Software (version 3.6.2; R Foundation for Statistical Computing, Vienna, Austria).

## Results

Associations of haplogroups with risk of clinical DLB and LBD-hDLB are detailed in Table 2. After adjusting for age and sex, no statistically significant associations were observed after Bonferroni correction (*P* <0.0024 considered significant). However, a nominally significant (*P* <0.05) association was reported between sub-haplogroup H and lower risk of clinical DLB (OR=0.61, *P*=0.006). This association was consistent when additionally adjusting for the number of apolipoprotein E4 (*APOE4*) alleles (OR=0.59, *P* = 0.004). No other associations approached statistical significance in any other series (all *P* ≥ 0.057, Table 2). Interestingly though, despite mitochondrial sub-haplogroup H not being strongly associated with LBD-hDLB (OR=0.87, *P* = 0.34), the protective association observed in the clinical DLB series was almost nominally significant when examining the combined DLB series (OR=0.78, *P* = 0.057) (Table 2).

In an exploratory analysis, we also evaluated associations of haplogroups with disease risks separately for males and females (Additional File 1: Tables S1 and S2). The aforementioned protective association between sub-haplogroup H and clinical DLB was observed relatively consistently in males (OR=0.64, *P* = 0.042) and females (OR=0.56, *P* = 0.066). Also, haplogroup HV/HV0a was suggestively associated with an increased risk of LBD-hDLB in males (OR=3.33, *P* = 0.044) and haplogroup V suggested an association with increased risk of both clinical DLB (OR = 4.29, *P* = 0.009) and overall DLB (OR = 3.56, *P* = 0.006) in females.

Associations of individual mitochondrial haplogroups with putaminal TH-ir and SN neuronal loss (Table 3), cortical Lewy body counts (Table 4), and diffuse LBD subtype (Additional File1: Table S3) were also assessed. No statistically significant associations were observed after correcting for multiple testing. A nominally significant association was noted between sub-haplogroup H and a less severe ventrolateral SN neuronal loss score (OR = 0.44, *P* = 0.033, Table 3) and also between sub-haplogroup T2 and a lower superior temporal LB count (multiplicative effect: 0.76, *P* = 0.044, Table 4). Both nominally significant associations remained consistent when additionally adjusting for the number of *APOE* ε4 alleles (*P* = 0.033 and *P* = 0.036 respectively). No associations between super-haplogroups and either risk of DLB or neuropathological outcomes were observed (all *P* ≥ 0.21).

**Table 3:** Associations of haplogroups with putaminal TH-ir (dorsolateral and ventromedial) and substantia nigra neuronal loss (ventrolateral and medial)

Mitochondrial DNA Haplogroup	Haplogroup frequency, No. (%), N = 242	Association with dorsolateral putaminal TH−ir		Association with ventromedial putaminal TH−ir		Association with ventrolateral substantia nigra neuronal loss score		Association with medial substantia nigra neuronal loss score	
		Regression coefficient (95% CI)	*P*−value	Regression coefficient (95% CI)	*P*−value	OR (95% CI)	*P*-value	OR (95% CI)	*P*-value
N^a^	0 (0.0%)	–	–	–	–	–	–	–	–
N1^a^	0 (0.0%)	–	–	–	–	–	–	–	–
I^a^	8 (3.3%)	–	–	–	–	–	–	–	–
W^a^	2 (0.8%)	–	–	–	–	–	–	–	–
X^a^	4 (1.7%)	–	–	–	–	–	–	–	–
R and R0^a^	1 (0.4%)	–	–	–	–	–	–	–	–
HV and HV0a^a^	8 (3.3%)	–	–	–	–	–	–	–	–
H, H1, H2, H3 and H4	91 (37.6%)	−0.05 (−0.15, 0.05)	0.32	−0.11 (−0.40, 0.18)	0.45	0.80 (0.43, 1.46)	0.46	1.12 (0.63, 1.97)	0.70
H	40 (16.5%)	0.05 (−0.09, 0.18)	0.51	0.11 (−0.27, 0.49)	0.55	0.44 (0.21, 0.93)	0.033	0.54 (0.26, 1.10)	0.090
H1	27 (11.2%)	−0.07 (−0.23, 0.09)	0.42	−0.13 (−0.58, 0.32)	0.57	1.33 (0.48, 3.68)	0.58	1.28 (0.52, 3.13)	0.59
H2^a^	8 (3.3%)	–	–	–	–	–	–	–	–
H3^a^	12 (5.0%)	−0.04 (−0.26, 0.19)	0.75	−0.29 (−0.92, 0.34)	0.36	0.90 (0.25, 3.31)	0.88	–	–
H4^a^	4 (1.7%)	–	–	–	–	–	–	–	–
V^a^	8 (3.3%)	–	–	–	–	–	–	–	–
JT^a^	0 (0.0%)	–	–	–	–	–	–	–	–
J, J1, J1d, J2a and J2b	26 (10.7%)	0.06 (−0.09, 0.21)	0.42	0.05 (−0.37, 0.47)	0.81	0.87 (0.36, 2.09)	0.75	0.62 (0.28, 1.39)	0.25
J^a^	0 (0.0%)	–	–	–	–	–	–	–	–
J1	23 (9.5%)	0.07 (−0.09, 0.22)	0.39	0.06 (−0.38, 0.50)	0.78	0.93 (0.37, 2.33)	0.87	0.72 (0.31, 1.69)	0.46
J1^a^d	0 (0.0%)	–	–	–	–	–	–	–	–
J2a^a^	2 (0.8%)	–	–	–	–	–	–	–	–
J2b^a^	1 (0.4%)	–	–	–	–	–	–	–	–
T, T1 and T2	30 (12.4%)	−0.03 (−0.19, 0.13)	0.70	−0.07 (−0.52, 0.38)	0.74	1.42 (0.49, 4.11)	0.52	2.60 (0.87, 7.81)	0.088
T1	0 (0.0%)	–	–	–	–	–	–	–	–
T1^a^	4 (1.7%)	–	–	–	–	–	–	–	–
T2	26 (10.7%)	0.02 (−0.15, 0.19)	0.82	0.14 (−0.35, 0.63)	0.58	1.35 (0.42, 4.30)	0.62	2.52 (0.72, 8.80)	0.15
U, U1, U3, U5, U6 and U8b'c	41 (16.9%)	−0.05 (−0.18, 0.08)	0.47	−0.15 (−0.53, 0.23)	0.44	1.75 (0.72, 4.24)	0.21	1.21 (0.57, 2.55)	0.62
U1	12 (5.0%)	–	–	–	–	–	–	–	–
U1^a^	2 (0.8%)	–	–	–	–	–	–	–	–
U3^a^	1 (0.4%)	–	–	–	–	–	–	–	–
U5	25 (10.3%)	−0.04 (−0.20, 0.12)	0.66	−0.17 (−0.62, 0.28)	0.45	1.59 (0.56, 4.51)	0.39	1.41 (0.56, 3.53)	0.47
U6^a^	0 (0.0%)	–	–	–	–	–	–	–	–
U8b'c^a^	1 (0.4%)	–	–	–	–	–	–	–	–
K	23 (9.5%)	0.00 (−0.18, 0.17)	0.98	0.04 (−0.45, 0.54)	0.87	0.60 (0.23, 1.59)	0.31	1.29 (0.49, 3.39)	0.61

For associations with putaminal TH-ir, regression coefficients, 95% CIs, and p-values result from linear regression models that were adjusted for age at death and sex, where due to their skewed distributions, lateral putaminal TH-ir was considered on the logarithm (base-10) scale and medial putaminal TH-ir was considered on the square root scale. Regression coefficients are interpreted as the additive increase on the mean outcome measure (on the logarithm or square root scale) for the given haplogroup. For associations with ventrolateral and medial substantia nigra neuronal loss scores, ORs, 95% CIs, and p-values result from proportional odds logistic regression models; ORs are interpreted at the multiplicative increase on the odds or a more severe neuronal loss score for the given haplogroup. After applying a Bonferroni correction for multiple testing separately for each outcome measure, *P*-values < 0.0045 (associations with lateral putaminal TH-ir, medial putaminal TH-ir, and ventrolateral substantia nigra neuronal loss score) and < 0.0050 (association with medial substantia nigra neuronal loss score) were considered statistically significant.

^a^Haplogroups that occurred in < 10 subjects in a given association analysis not examined in that analysis. TH-ir=tyrosine hydroxylase immunoreactivity; CI = confidence interval; OR = odds ratio.

**Table 4:** Associations of mitochondrial DNA haplogroups with cortical Lewy body counts

Mitochondrial DNA Haplogroup	Haplogroup frequency, No. (%), N = 242	Association with middle frontal LB count		Association with superior temporal LB count		Association with inferior parietal LB count		Association with cingulate LB count		Association with parahippocampal LB count	
		Multiplicative effect (95% CI)	*P*−value	Multiplicative effect (95% CI)	*P*−value	Multiplicative effect (95% CI)	*P*-value	Multiplicative effect (95% CI)	*P*-value	Multiplicative effect (95% CI)	*P*-value
N^a^	0 (0.0%)	–	–	–	–	–	–	–	–	–	–
N1^a^	0 (0.0%)	–	–	–	–	–	–	–	–	–	–
I^a^	8 (3.3%)	–	–	–	–	–	–	–	–	–	–
W^a^	2 (0.8%)	–	–	–	–	–	–	–	–	–	–
X^a^	4 (1.7%)	–	–	–	–	–	–	–	–	–	–
R and R0^a^	1 (0.4%)	–	–	–	–	–	–	–	–	–	–
HV and HV0a^a^	8 (3.3%)	–	–	–	–	–	–	–	–	–	–
H, H1, H2, H3 and H4	91 (37.6%)	1.16 (0.95, 1.43)	0.15	1.05 (0.89, 1.25)	0.56	1.12 (0.91, 1.39)	0.28	1.06 (0.92, 1.22)	0.41	1.02 (0.88, 1.18)	0.83
H	40 (16.5%)	1.01 (0.77, 1.34)	0.93	0.93 (0.74, 1.18)	0.57	0.89 (0.67, 1.18)	0.41	0.94 (0.78, 1.14)	0.54	0.96 (0.80, 1.17)	0.70
H1	27 (11.2%)	1.10 (0.80, 1.52)	0.57	0.94 (0.72, 1.24)	0.64	1.08 (0.78, 1.50)	0.64	1.00 (0.80, 1.25)	0.98	0.90 (0.72, 1.13)	0.34
H2^a^	8 (3.3%)	–	–	–	–	–	–	–	–	–	–
H3	12 (5.0%)	1.03 (0.66, 1.65)	0.91	1.18 (0.82, 1.75)	0.38	1.22 (0.78, 1.94)	0.40	1.10 (0.82, 1.51)	0.52	1.03 (0.76, 1.42)	0.84
H4^a^	4 (1.7%)	–	–	–	–	–	–	–	–	–	–
V^a^	8 (3.3%)	–	–	–	–	–	–	–	–	–	–
JT^a^	0 (0.0%)	–	–	–	–	–	–	–	–	–	–
J, J1, J1d, J2a and J2b	26 (10.7%)	0.92 (0.67, 1.30)	0.64	0.99 (0.76, 1.31)	0.94	1.13 (0.81, 1.58)	0.48	0.99 (0.79, 1.25)	0.92	1.02 (0.81, 1.31)	0.85
J^a^	0 (0.0%)	–	–	–	–	–	–	–	–	–	–
J1	23 (9.5%)	0.87 (0.61, 1.25)	0.44	0.96 (0.72, 1.29)	0.77	1.02 (0.71, 1.47)	0.92	0.97 (0.76, 1.24)	0.79	1.03 (0.81, 1.34)	0.80
J1d^a^	0 (0.0%)	–	–	–	–	–	–	–	–	–	–
J2a^a^	2 (0.8%)	–	–	–	–	–	–	–	–	–	–
J2b^a^	1 (0.4%)	–	–	–	–	–	–	–	–	–	–
T, T1 and T2	30 (12.4%)	1.05 (0.78, 1.43)	0.75	0.81 (0.63, 1.06)	0.11	0.88 (0.64, 1.21)	0.42	0.95 (0.77, 1.16)	0.59	0.91 (0.74, 1.12)	0.37
T^a^	0 (0.0%)	–	–	–	–	–	–	–	–	–	–
T1^a^	4 (1.7%)	–	–	–	–	–	–	–	–	–	–
T2	26 (10.7%)	1.03 (0.75, 1.43)	0.86	0.76 (0.58, 1.00)	0.044	0.79 (0.57, 1.12)	0.18	0.93 (0.75, 1.16)	0.50	0.90 (0.72, 1.13)	0.34
U, U1, U3, U5, U6 and U8b'c	41 (16.9%)	0.84 (0.64, 1.10)	0.20	1.02 (0.82, 1.28)	0.85	0.91 (0.69, 1.20)	0.49	0.97 (0.81, 1.16)	0.70	0.97 (0.80, 1.18)	0.75
U	12 (5.0%)	0.94 (0.60, 1.52)	0.79	1.16 (0.80, 1.71)	0.45	0.95 (0.59, 1.55)	0.82	1.11 (0.82, 1.52)	0.49	–	–
U1^a^	2 (0.8%)	–	–	–	–	–	–	–	–	–	–
U3^a^	1 (0.4%)	–	–	–	–	–	–	–	–	–	–
U5	25 (10.3%)	0.82 (0.59, 1.15)	0.23	0.95 (0.72, 1.25)	0.69	0.95 (0.68, 1.33)	0.74	0.91 (0.73, 1.14)	0.40	0.92 (0.72, 1.18)	0.50
U6^a^	0 (0.0%)	–	–	–	–	–	–	–	–	–	–
U8b'c^a^	1 (0.4%)	–	–	–	–	–	–	–	–	–	–
K	23 (9.5%)	1.15 (0.82, 1.64)	0.43	1.17 (0.88, 1.57)	0.30	1.10 (0.77, 1.59)	0.60	1.06 (0.84, 1.35)	0.64	1.13 (0.87, 1.48)	0.36

Multiplicative effects, 95% CIs, and p-values result from negative binomial regression models that were adjusted for age at death and sex. Multiplicative effects are interpreted as the multiplicative increase on the mean LB count for the given haplogroup. After applying a Bonferroni correction for multiple testing separately for each outcome measure, *P*-values < 0.0042 (associations with middle frontal, superior temporal, inferior parietal, and cingulate LB counts) and < 0.0045 (associations with parahippocampal LB count) were considered statistically significant.

^a^Haplogroups that occurred in < 10 subjects in a given association analysis not examined in that analysis. LB = Lewy body. CI = confidence interval

## Discussion

Efforts to understand the genetic etiology of DLB have identified shared genetic markers between PD and AD [3, 23, 27, 34]. Physiologically, mitochondrial dysfunction is consistently reported in synucleinopathies [32] and mitochondrial phenotypes are predisposed by variation in mtDNA [15, 16]. Interestingly, LB pathology is more prevalent in older individuals with mitochondrial disease compared to controls [9], further emphasizing the importance of mtDNA in disease pathology.

Acknowledging this, our examinations of mtDNA variation, in the form of mitochondrial haplogroups, with DLB risk and neuropathological measures in this study reported no statistically significant associations after applying Bonferroni correction. However, mtDNA sub-haplogroup H reported a suggestive protective effect with clinical DLB risk (OR=0.61, *P* = 0.006) which was not observed in LBD-hDLB cases (OR=0.87, *P* = 0.34). Interestingly though, sub-haplogroup H also indicated an association with less severe ventrolateral SN neuronal loss (OR=0.44, *P* = 0.033) in LBD-hDLB cases.

Mitochondrial haplogroup H is the most common haplogroup in European populations, accounting for more than 40% of individuals [45]. Mitochondrial haplogroup H has more than 80 sub-haplogroups which are predominantly defined by variation in mtDNA coding regions [48], with sub-haplogroups H1 and H3 being the most common. Mitochondrial haplogroup H has previously been associated with increased risk of PD [21] and DLB [5]. Notably, the results reported in Hudson and colleagues’ study was not an exact association of haplogroup H with PD risk, as they grouped haplogroup H and haplogroup V cases together. Although this was statistically more reliable, genetically the results do not clarify what mtDNA variants are driving PD risk because haplogroups H and V are phylogenetically related but are genetically different. Moreover, Chinnery *et al.* reported increased risk of haplogroup H with DLB in only 84 DLB cases and did not evaluate H sub-haplogroups. In this study, we assessed associations between mtDNA haplogroups and sub-haplogroups with DLB risk more comprehensively and in a much larger DLB cohort. It is possible that the elevated risk of DLB with mtDNA haplogroup H background reported by Chinnery *et al.* may be an artefact of common H sub-haplogroups, such as H1 and H3, inducing more detrimental risk outcomes with DLB—which is also observed in our data. Overall, these studies demonstrate the functional heterogeneity even within a given haplogroup and reinforce the need to stratify haplogroups into sub-haplogroups in genetic studies [13]. Replication will be important to validate our findings.

Disappointingly, we did not replicate the sub-haplogroup H association with reduced DLB risk in pathologically confirmed LBD-hDLB cases, nor in the combined cases group. This may be because one cohort was clinically defined whereas the other cohort was neuropathologically defined. Neuropathologically confirmed cases were included in this study if they were deemed as having a high likelihood of clinical dementia—which was determined from pathology propensity in cortical regions and available medical records. It is possible that the neuropathologically defined LBD-hDLB cohort may also contain clinical PDD cases. PDD has a different disease course to DLB and is diagnosed when dementia develops more than a year after parkinsonism onset and can be considered a much slower progression of dementia than DLB [30]. Interestingly, this data suggests that mitochondrial sub-haplogroup H may be protective against DLB but not PDD, which may suggest that mitochondrial background influences rate of dementia progression in PD.

Interestingly, we did observe a suggestive association between mtDNA sub-haplogroup H background and less severe ventrolateral SN neuronal loss in LBD-hDLB cases. Albeit not statistically significant, this data is important because SN degeneration is a classical hallmark of PD and may behave as an important mediator in LB spread in LBD and may be a defining mediator between DLB and PDD. This concept also supports the rationale that sub-haplogroup H may be protective against DLB risk, as reported in this study. Functionally this could be explained by SN cells being more sensitive to physiological pressures than other neuronal types. More specifically, dopaminergic SN cells are highly metabolically active, long and thin, and have little to no myelination [2], and they heavily rely on healthy mitochondria for efficient OXPHOS to ensure sufficient ATP is produced to maintain their metabolic capacity. Mitochondria carrying mtDNA haplogroup H are reported to have the most efficient OXPHOS coupling capacity in all European haplogroups and produce more ATP and ROS than other groups [50]. This may be advantageous in protecting SN cells from accumulating LB with age. On the contrary though, SN cells with a haplogroup H background may be more susceptible to physiological pressures as functional studies in cybrid cell lines have demonstrated these cells have an increased susceptibility to oxidative stress compared to non-haplogroup H cells [31]. This suggests that mitochondrial background may provide cell or regional-specific biological benefits, but under additional physiological pressures may enhance disease progression.

As LBD pathology is very heterogenous and presents with pathological aggregates of tau, beta-amyloid, and TDP-43 proteins, it is important to also consider the role nuclear genetic risk factors play in driving disease risk relative to mtDNA background. More specifically, *APOE4* is consistently an increased genetic risk factor for clinical DLB [18, 40] and AD [23, 41], and *APOE4* influences LB pathology independently to AD pathology [8]. The mtDNA haplogroup associations reported in this study were all adjusted for *APOE4* allele status which did not change any observations after adjustments. Reassuringly, mtDNA haplogroup associations in DLB and AD have been reported independent of *APOE4* status in prior studies [5], suggesting both mitochondrial and nuclear genomic background influence disease phenotypes. Future studies should consider evaluating major nuclear genetic risk factors relative to mitochondrial genetic background to avoid any possible bias.

Several limitations of our study are important to note. The main limitation being that even though the sample size of DLB and LBD-hDLB cases are relatively large given the prevalence of DLB, sample numbers are small for a genetic association study and therefore the possibility of a type II error is important to consider. This is especially true when considering adjustment for multiple testing and for rare haplogroups. In addition, although all cases in this study were examined for population stratification prior to conducting this work [3]; population stratification in the control cohort may influence false positive findings (noting all subjects carried European mtDNA haplogroups). Global access to well described cohorts of DLB cases is required and validation of this work in larger cohorts of DLB and LBD-hDLB cases will be important to further investigate the role mitochondrial haplogroup H has in DLB risk and neuropathological development.

## Conclusions

We have conducted a comprehensive study of the role of mitochondrial genomic variation, in the form of mitochondrial haplogroups and sub-haplogroups, in clinical DLB and pathologically confirmed LBD-hDLB. Moreover, this is one of the first studies to explore the association of mtDNA background with neuropathological LB counts and neuronal loss measures in LBD-hDLB brains. Our data suggests that mitochondrial sub-haplogroup H may be protective against clinical DLB risk, independent of *APOE4* background, and this may be indirectly influenced by the suggestive association that sub-haplogroup H is protective against neuronal loss in substantia nigra tissue. Additional assessments and replication studies are warranted to further validate and expand on this data.

## Supplementary Information

**Additional file 1.** Supplementary Tables.

## Data Availability

The datasets that were used and analyzed during the current study are available from the corresponding author on reasonable request.

## Abbreviations

AD: Alzheimer’s disease
APOE4: Apolipoprotein E4 allele
aSyn: Alpha-synuclein
ATP: Adenosine triphosphate
CIs: Confidence intervals
DLB: Dementia with Lewy bodies
DNA: Deoxyribonucleic acid
LB: Lewy bodies
LBD: Lewy body disease
LBD-hDLB: Lewy body disease with a high likelihood of having clinical DLB
mtDNA: Mitochondrial DNA
NFTs: Neurofibrillary tangles
ORs: Odds ratios
OXPHOS: Oxidative phosphorylation
PDD: Parkinson’s disease dementia
ROS: Reactive oxygen species
SN: Substantia nigra
SP: Senile plaques
TH-ir: Tyrosine hydroxylase immunoreactivity
