# Supplementary material for: Mitochondrial genomic variation in dementia with Lewy bodies: association with disease risk and neuropathological measures
Source: Acta Neuropathol Commun. 2022 Jul 14;10:103. doi: 10.1186/s40478-022-01399-4 (PMC9281088; doi:10.1186/s40478-022-01399-4)
Supplement: Supplementary file 1 — Additional file 1. Supplementary Tables. [file 40478_2022_1399_MOESM1_ESM.pdf]

Supplementary Tables

	Haplogroup frequency, No. (%)				Clinical DLB vs. controls		LBD with a high likelihood of DLB vs. controls		Overall DLB vs. controls	
**Mitochondrial DNA Haplogroup**	Controls (N=388)	Clinical DLB (N=270)	LBD high likelihood of DLB (N=292)	Overall DLB (N=523)	OR (95% CI)	P-value	OR (95% CI)	P-value	OR (95% CI)	P-value
**N[1]**	0 (0.0%)	0 (0.0%)	0 (0.0%)	0 (0.0%)	---	---	---	---	---	---
**N1[1]**	2 (0.5%)	4 (1.5%)	1 (0.3%)	5 (1.0%)	---	---	---	---	---	---
**I**	16 (4.1%)	10 (3.7%)	8 (2.7%)	15 (2.9%)	0.82 (0.36, 1.89)	0.65	0.63 (0.27, 1.50)	0.30	0.63 (0.30, 1.31)	0.22
**W**	6 (1.5%)	6 (2.2%)	5 (1.7%)	9 (1.7%)	1.39 (0.43, 4.46)	0.58	1.11 (0.33, 3.67)	0.87	1.09 (0.38, 3.10)	0.88
**X**	5 (1.3%)	1 (0.4%)	4 (1.4%)	5 (1.0%)	---	---	---	---	0.75 (0.21, 2.62)	0.65
**R or R0[1]**	3 (0.8%)	1 (0.4%)	1 (0.3%)	2 (0.4%)	---	---	---	---	---	---
**HV or HV0a**	4 (1.0%)	6 (2.2%)	10 (3.4%)	14 (2.7%)	2.01 (0.56, 7.27)	0.29	3.33 (1.03, 10.73)	0.044	2.52 (0.82, 7.75)	0.11
**H, H1, H2, H3, and H4**	174 (44.8%)	110 (40.7%)	115 (39.4%)	213 (40.7%)	0.87 (0.63, 1.20)	0.40	0.81 (0.59, 1.10)	0.17	0.86 (0.66, 1.12)	0.27
**H**	78 (20.1%)	38 (14.1%)	57 (19.5%)	92 (17.6%)	0.64 (0.41, 0.98)	0.042	0.96 (0.66, 1.41)	0.84	0.85 (0.60, 1.19)	0.33
**H1**	63 (16.2%)	50 (18.5%)	30 (10.3%)	75 (14.3%)	1.23 (0.81, 1.88)	0.33	0.60 (0.38, 0.96)	0.031	0.88 (0.61, 1.28)	0.51
**H2**	15 (3.9%)	3 (1.1%)	9 (3.1%)	12 (2.3%)	0.29 (0.08, 1.05)	0.058	0.79 (0.34, 1.84)	0.59	0.61 (0.28, 1.34)	0.22
**H3**	12 (3.1%)	12 (4.4%)	13 (4.5%)	23 (4.4%)	1.44 (0.63, 3.31)	0.39	1.48 (0.66, 3.28)	0.34	1.44 (0.70, 2.94)	0.32
**H4**	6 (1.5%)	7 (2.6%)	6 (2.1%)	11 (2.1%)	1.76 (0.57, 5.40)	0.32	1.36 (0.43, 4.25)	0.60	1.38 (0.50, 3.79)	0.53
**V**	10 (2.6%)	8 (3.0%)	6 (2.1%)	14 (2.7%)	1.20 (0.46, 3.18)	0.71	0.78 (0.28, 2.18)	0.64	1.03 (0.45, 2.36)	0.95
**JT[1]**	0 (0.0%)	0 (0.0%)	0 (0.0%)	0 (0.0%)	---	---	---	---	---	---
**J, J1, J1d, J2, J2a, and J2b**	44 (11.3%)	27 (10.0%)	33 (11.3%)	59 (11.3%)	0.88 (0.52, 1.48)	0.63	1.01 (0.62, 1.63)	0.98	1.00 (0.66, 1.52)	0.99
**J**	0 (0.0%)	0 (0.0%)	0 (0.0%)	0 (0.0%)	---	---	---	---	---	---
**J1**	36 (9.3%)	18 (6.7%)	28 (9.6%	45 (8.6%)	0.71 (0.39, 1.30)	0.27	1.05 (0.62, 1.76)	0.86	0.94 (0.59, 1.49)	0.79
**J1d**	1 (0.3%)	1 (0.4%)	0 (0.0%)	1 (0.2%)	---	---	---	---	---	---
**J2a**	3 (0.8%)	5 (1.9%)	4 (1.4%)	9 (1.7%)	---	---	---	---	2.26 (0.60, 8.49)	0.23
**J2b**	4 (1.0%)	3 (1.1%)	1 (0.3%)	4 (0.8%)	---	---	---	---	---	---
**T, T1, and T2**	30 (7.7%)	23 (8.2%)	29 (9.9%)	47 (9.0%)	1.13 (0.63, 2.03)	0.67	1.30 (0.76, 2.22)	0.34	1.16 (0.72, 1.89)	0.54
**T[1]**	0 (0.0%)	0 (0.0%)	0 (0.0%)	0 (0.0%)	---	---	---	---	---	---
**T1**	10 (2.6%)	3 (1.1%)	6 (2.1%)	9 (1.7%)	0.39 (0.10, 1.47)	0.16	0.77 (0.28, 2.15)	0.62	0.63 (0.25, 1.59)	0.33
**T2**	20 (5.2%)	20 (7.4%)	23 (7.9%)	38 (7.3%)	1.55 (0.81, 2.98)	0.19	1.56 (0.84, 2.91)	0.16	1.44 (0.82, 2.53)	0.20
**U, U1, U3, U5, U6, and U8b'c**	60 (15.5%)	39 (14.4%)	50 (17.1%)	81 (15.5%)	0.88 (0.56, 1.37)	0.57	1.12 (0.74, 1.69)	0.59	0.99 (0.69, 1.43)	0.95
**U**	21 (5.4%)	19 (7.0%)	18 (6.2%)	34 (6.5%)	1.31 (0.68, 2.52)	0.42	1.15 (0.60, 2.21)	0.67	1.22 (0.70, 2.15)	0.49
**U1[1]**	1 (0.3%)	0 (0.0%)	0 (0.0%)	0 (0.0%)	---	---	---	---	---	---
**U3[1]**	3 (0.8%)	0 (0.0%)	1 (0.3%)	1 (0.2%)	---	---	---	---	---	---
**U5**	35 (9.0%)	20 (7.4%)	30 (10.3%)	45 (8.6%)	0.75 (0.42, 1.35)	0.34	1.14 (0.68, 1.90)	0.63	0.93 (0.58, 1.48)	0.75
**U6[1]**	0 (0.0%)	0 (0.0%)	0 (0.0%)	0 (0.0%)	---	---	---	---	---	---
**U8b'c[1]**	0 (0.0%)	0 (0.0%)	1 (0.3%)	1 (0.2%)	---	---	---	---	---	---
**K**	34 (8.8%)	35 (13.0%)	30 (10.3%)	59 (11.3%)	1.51 (0.90, 2.52)	0.12	1.20 (0.71, 2.01)	0.49	1.33 (0.85, 2.08)	0.21

Supplementary Table 1: Associations between mitochondrial DNA haplogroups and risk of DLB in males. OR=odds ratio; CI=confidence interval. ORs, 95% CIs, and p-values result from logistic regression models that were adjusted for age. The cases that were in both the clinical DLB series and the LBD with a high likelihood of DLB

series were included only once in the overall DLB series. After applying a Bonferroni correction for multiple testing, p-values <0.0026 (clinical DLB vs. controls), <0.0026 (LBD with a high likelihood of DLB vs. controls), and <0.0024 (overall DLB vs. controls) were considered as statistically significant. [1]Haplogroups that occurred in <10 subjects in a given association analysis were not examined.

	Haplogroup frequency, No. (%)				Clinical DLB vs. controls		LBD with a high likelihood of DLB vs. controls		Overall DLB vs. controls	
**Mitochondrial DNA Haplogroup**	Controls (N=522)	Clinical DLB (N=90)	LBD high likelihood of DLB (N=154)	Overall DLB (N=235)	OR (95% CI)	P-value	OR (95% CI)	P-value	OR (95% CI)	P-value
**N[1]**	2 (0.4%)	0 (0.0%)	0 (0.0%)	0 (0.0%)	---	---	---	---	---	---
**N1[1]**	3 (0.6%)	0 (0.0%)	1 (0.6%)	1 (0.4%)	---	---	---	---	---	---
**I**	15 (2.9%)	2 (2.2%)	5 (3.2%)	7 (3.0%)	0.67 (0.15, 3.01)	0.60	1.27 (0.45, 3.61)	0.65	1.08 (0.43, 2.70)	0.87
**W**	9 (1.7%)	3 (3.3%)	3 (1.9%)	6 (2.6%)	2.12 (0.56, 8.04)	0.27	1.03 (0.27, 3.87)	0.97	1.46 (0.51, 4.16)	0.48
**X**	3 (0.6%)	1 (1.1%)	3 (1.9%)	4 (1.7%)	---	---	---	---	---	---
**R or R0[1]**	3 (0.6%)	0 (0.0%)	0 (0.0%)	0 (0.0%)	---	---	---	---	---	---
**HV or HV0a**	18 (3.4%)	0 (0.0%)	3 (1.9%)	3 (1.3%)	N/A[1]	0.091	0.52 (0.15, 1.79)	0.30	0.35 (0.10, 1.20)	0.095
**H, H1, H2, H3, and H4**	249 (47.7%)	39 (43.3%)	68 (44.2%)	103 (43.8%)	0.83 (0.53, 1.30)	0.41	0.88 (0.61, 1.27)	0.51	0.86 (0.63, 1.17)	0.34
**H**	121 (23.2%)	13 (14.4%)	28 (18.2%)	41 (17.4%)	0.56 (0.30, 1.04)	0.066	0.73 (0.46, 1.16)	0.18	0.70 (0.47, 1.04)	0.076
**H1**	82 (15.7%)	17 (18.9%)	26 (16.9%)	41 (17.4%)	1.23 (0.69, 2.19)	0.49	1.13 (0.69, 1.83)	0.63	1.14 (0.76, 1.73)	0.52
**H2**	21 (4.0%)	3 (3.3%)	3 (1.9%)	5 (2.1%)	0.78 (0.23, 2.68)	0.69	0.52 (0.15, 1.77)	0.30	0.54 (0.20, 1.44)	0.22
**H3**	20 (3.8%)	4 (4.4%)	10 (6.5%)	13 (5.5%)	1.28 (0.42, 3.85)	0.67	1.62 (0.74, 3.56)	0.23	1.42 (0.69, 2.91)	0.34
**H4**	5 (1.0%)	2 (2.2%)	1 (0.6%)	3 (1.3%)	---	---	---	---	---	---
**V**	8 (1.5%)	6 (6.7%)	6 (3.9%)	12 (5.1%)	4.29 (1.45, 12.75)	0.009	2.77 (0.93, 8.20)	0.066	3.56 (1.43, 8.86)	0.006
**JT[1]**	2 (0.4%)	0 (0.0%)	0 (0.0%)	0 (0.0%)	---	---	---	---	---	---
**J, J1, J1d, J2, J2a, and J2b**	49 (9.4%)	11 (12.2%)	19 (12.3%)	28 (11.9%)	1.36 (0.68, 2.73)	0.39	1.35 (0.77, 2.39)	0.30	1.31 (0.80, 2.14)	0.29
**J**	0 (0.0%)	1 (1.1%)	0 (0.0%)	1 (0.4%)	---	---	---	---	---	---
**J1**	36 (6.9%)	7 (7.8%)	13 (8.4%)	19 (8.1%)	1.15 (0.49, 2.67)	0.75	1.27 (0.65, 2.47)	0.49	1.20 (0.67, 2.13)	0.55
**J1d**	0 (0.0%)	0 (0.0%)	0 (0.0%)	0 (0.0%)	---	---	---	---	---	---
**J2a**	10 (1.9%)	2 (2.2%)	5 (3.2%)	6 (2.6%)	1.2 (0.26, 5.62)	0.81	1.66 (0.56, 4.99)	0.36	1.32 (0.47, 3.67)	0.60
**J2b**	3 (0.6%)	1 (1.1%)	1 (0.6%)	2 (0.9%)	---	---	---	---	---	---
**T, T1, and T2**	47 (9.0%)	9 (10.0%)	16 (10.4%)	24 (10.2%)	1.17 (0.55, 2.49)	0.68	1.11 (0.60, 2.02)	0.75	1.13 (0.67, 1.90)	0.65
**T[1]**	0 (0.0%)	1 (1.1%)	0 (0.0%)	1 (0.4%)	---	---	---	---	---	---
**T1**	7 (1.3%)	2 (2.2%)	2 (1.3%)	4 (1.7%)	---	---	---	---	1.23 (0.36, 4.26)	0.74
**T2**	40 (7.7%)	6 (6.7%)	14 (9.1%)	19 (8.1%)	0.90 (0.37, 2.19)	0.81	1.15 (0.60, 2.18)	0.68	1.04 (0.59, 1.84)	0.89
**U, U1, U3, U5, U6, and U8b'c**	70 (13.4%)	14 (15.6%)	17 (11.0%)	29 (12.3%)	1.19 (0.63, 2.22)	0.59	0.81 (0.46, 1.42)	0.46	0.91 (0.57, 1.45)	0.70
**U**	23 (4.4%)	3 (3.3%)	3 (1.9%)	6 (2.6%)	0.73 (0.21, 2.49)	0.61	0.44 (0.13, 1.50)	0.19	0.58 (0.23, 1.43)	0.24
**U1[1]**	0 (0.0%)	0 (0.0%)	2 (1.3%)	2 (0.9%)	---	---	---	---	---	---
**U3[1]**	5 (1.0%)	0 (0.0%)	0 (0.0%)	0 (0.0%)	---	---	---	---	---	---
**U5**	39 (7.5%)	10 (11.1%)	12 (7.8%)	20 (8.5%)	1.58 (0.75, 3.29)	0.23	1.03 (0.52, 2.02)	0.94	1.15 (0.65, 2.01)	0.63
**U6[1]**	3 (0.6%)	1 (1.1%)	0 (0.0%)	1 (0.4%)	---	---	---	---	---	---
**U8b'c[1]**	0 (0.0%)	0 (0.0%)	0 (0.0%)	0 (0.0%)	---	---	---	---	---	---
**K**	44 (8.2%)	5 (5.6%)	13 (8.4%)	18 (7.7%)	0.65 (0.25, 1.69)	0.38	1.01 (0.52, 1.93)	0.99	0.90 (0.51, 1.59)	0.71

Supplementary Table 2: Associations between mitochondrial DNA haplogroups and risk of DLB in females. OR=odds ratio; CI=confidence interval. ORs, 95% CIs, and p-values result from logistic regression models that were adjusted for age and sex. The cases that were in both the clinical DLB series and the LBD with a high likelihood of DLB series were included only once in the overall DLB series. [1] Logistic regression analysis was not possible owing to a zero cell count; the p-value results from Fisher's exact test. After applying a Bonferroni correction for multiple testing, p-values <0.0028 (clinical DLB vs. controls), <0.0028 (LBD with a high likelihood of DLB

vs. controls), and <0.0026 (overall DLB vs. controls) were considered as statistically significant. [1]Haplogroups that occurred in <10 subjects in a given association analysis were not examined.

		Association with diffuse LBD	
**Mitochondrial DNA Haplogroup**	**Haplogroup frequency, No. (%), N=446**	**OR (95% CI)**	**P-value**
N[1]	0 (0.0%)	---	---
└ N1[1]	2 (0.4%)	---	---
└ I	13 (2.9%)	0.57 (0.16, 1.96)	0.37
└ W[1]	8 (1.8%)	---	---
└ X[1]	7 (1.6%)	---	---
└ R and R0[1]	1 (0.2%)	---	---
└ HV and HV0a	13 (2.9%)	0.89 (0.24, 3.40)	0.87
└ H, H1, H2, H3 and H4	183 (41.0%)	1.11 (0.68, 1.80)	0.68
└ H	85 (19.1%)	1.00 (0.55, 1.82)	0.99
└ H1	56 (12.6%)	1.21 (0.57, 2.61)	0.62
└ H2	12 (2.7%)	3.06 (0.39, 24.13)	0.29
└ H3	23 (5.2%)	0.79 (0.28, 2.22)	0.65
└ H4[1]	7 (1.6%)	---	---
└ V[2]	12 (2.7%)	N/A	0.14
└ JT[1]	0 (0.0%)	---	---
└ J, J1, J1d, J2a and J2b	52 (11.7%)	0.82 (0.40, 1.64)	0.57
└ J[1]	0 (0.0%)	---	---
└ J1	41 (9.2%)	0.67 (0.32, 1.42)	0.30
└ J1d[1]	0 (0.0%)	---	---
└ J2a[1]	9 (2.0%)	---	---
└ J2b[1]	2 (0.4%)	---	---
└ T, T1 and T2	45 (10.1%)	0.59 (0.29, 1.20)	0.14
└ T[1]	0 (0.0%)	---	---
└ T1[1]	8 (1.8%)	---	---
└ T2	37 (8.3%)	0.57 (0.26, 1.22)	0.15
└ U, U1, U3, U5, U6 and U8b'c	67 (15.0%)	0.87 (0.46, 1.65)	0.67
└ U	21 (4.7%)	0.62 (0.23, 1.68)	0.35

Mitochondrial DNA Haplogroup	Haplogroup frequency, No. (%), N=446	Association with diffuse LBD	
		OR (95% CI)	P-value
└ U1[1]	2 (0.4%)	---	---
└ U3[1]	1 (0.2%)	---	---
└ U5	42 (9.4%)	1.33 (0.56, 3.12)	0.52
└ U6[1]	0 (0.0%)	---	---
└ U8b'c[1]	1 (0.2%)	---	---
└ K	43 (9.6%)	1.57 (0.63, 3.86)	0.33

Supplementary Table 3: Associations between mitochondrial DNA haplogroups and diffuse Lewy body disease. ORs, 95% CIs, and p-values result from logistic regression models that were adjusted for age at death and sex. ORs are interpreted as the multiplicative increase on the diffuse LBD for the given haplogroup. After applying a Bonferroni correction for multiple testing, p-values <0.0031 were considered statistically significant. [1]Haplogroups that occurred in <10 subjects in a given association analysis were not examined. [2]Logistic regression was not possible for the V haplogroup owing to the fact that there were no transitional LBD cases who had the V haplogroup; therefore, the p-value results from Fisher's exact test. LBD=Lewy body disease; OR=odds ratio; CI=confidence interval.
